# Deciphering the Host–Pathogen Interactome of the Wheat–Common Bunt System: A Step towards Enhanced Resilience in Next Generation Wheat

**DOI:** 10.3390/ijms23052589

**Published:** 2022-02-26

**Authors:** Raghav Kataria, Rakesh Kaundal

**Affiliations:** 1 Department of Plants, Soils, and Climate, College of Agriculture and Applied Sciences, Utah State University, Logan, UT 84322, USA; raghav.kataria@usu.edu; 2 Bioinformatics Facility, Center for Integrated BioSystems, College of Agriculture and Applied Sciences, Utah State University, Logan, UT 84322, USA; 3 Department of Computer Science, College of Science, Utah State University, Logan, UT 84322, USA

**Keywords:** wheat, *Tilletia*, common bunt, host–pathogen interactions, effectors, secretory, R-genes, computational prediction

## Abstract

Common bunt, caused by two fungal species, *Tilletia caries* and *Tilletia laevis*, is one of the most potentially destructive diseases of wheat. Despite the availability of synthetic chemicals against the disease, organic agriculture relies greatly on resistant cultivars. Using two computational approaches—interolog and domain-based methods—a total of approximately 58 M and 56 M probable PPIs were predicted in *T. aestivum*–*T. caries* and *T. aestivum*–*T. laevis* interactomes, respectively. We also identified 648 and 575 effectors in the interactions from *T. caries* and *T. laevis*, respectively. The major host hubs belonged to the serine/threonine protein kinase, hsp70, and mitogen-activated protein kinase families, which are actively involved in plant immune signaling during stress conditions. The Gene Ontology (GO) and Kyoto Encyclopedia of Genes and Genomes (KEGG) enrichment analysis of the host proteins revealed significant GO terms (O-methyltransferase activity, regulation of response to stimulus, and plastid envelope) and pathways (NF-kappa B signaling and the MAPK signaling pathway) related to plant defense against pathogens. Subcellular localization suggested that most of the pathogen proteins target the host in the plastid. Furthermore, a comparison between unique *T. caries* and *T. laevis* proteins was carried out. We also identified novel host candidates that are resistant to disease. Additionally, the host proteins that serve as transcription factors were also predicted.

## 1. Introduction

Wheat (*Triticum aestivum* L.), belonging to the *Poaceae* family, is one of the most widely cultivated crop species. Being an important staple food, it feeds a large part of the world’s population. In terms of global production and plant acreage, wheat ranks third and comprises about 35% of the total food grain of the world [1]. It is considered as a primary source of carbohydrates, proteins, fiber, and various other minor nutrients, such as minerals, lipids, phytochemicals, and vitamins [2], which makes wheat one of the most favorable food grains worldwide. Wheat is widely grown in a broad range of environments, such as in temperate, tropical, warm/humid, or cold/dry conditions [3]. According to the recent (2021) assessment on wheat production by the FAO (Food and Agriculture Organization), there has been a significant yield gain in wheat production as compared to past years (http://www.fao.org/worldfoodsituation/csdb/en/, accessed on 05 May 2021); however, the increasing population and dietary changes of people are leading to the need for substantial yield gains in the future to meet this escalating demand [4].

Various factors, such as climate change and abiotic and biotic stresses, are a major threat to global wheat production. Diseases and pests contribute to about 20% of the loss of wheat production globally. Among the fungal pathogens, *Blumeria graminis*, *Puccinia* species, *Fusarium* species, and *Tilletia* species are considered as the most important pathogens, affecting the crop yield to a great extent [5]. Common bunt, the most potentially destructive disease of wheat, is caused by *Tilletia caries* (syn. *Tilletia tritici*) and *Tilletia laevis* (syn. *Tilletia foetida*). An outbreak of this disease has been observed in countries worldwide, including the United Kingdom [6], Czech Republic [7], Romania [8], and others. Both species are similar in their life cycle and germination and they produce similar disease symptoms [9]. The disease is seed-borne, typically induced by the teliospores of the fungi under favorable conditions, which contaminate the seed surface and infect the coleoptile before the seedling’s emergence [10]. Since the grains are replaced with unpleasant-smelling brown to black bunt ball spores, the infected plants lead to reduced grain yield and seed quality in comparison to a healthy plant [11]. Due to the production of trimethylamine, the infected spikes of wheat have a foul odor [12]. The disease can be effectively managed with the fungicidal treatment of the seeds; however, in the case of low-input agriculture, the wide usage of fungicides is not applicable [13].

Molecular interactions form the basis of pathogenicity [14]. Proteins interact with other proteins, either through transient or permanent protein complexes, in order to perform various essential biological functions. In systems biology, inferring the protein–protein interactions (PPIs) between host and pathogen form the basis of understanding for disease infection mechanisms [15]. The prediction of PPIs with the help of bioinformatics techniques enhances the study of protein function and cellular behavior [16]. A wide range of computational approaches, such as phylogenetic profiling, gene ontology (GO) annotation, protein domain interactions, sequence similarity, and others, have been developed for the prediction of host–pathogen interactions (HPIs). Among the available methods, the sequence-based and domain-based approaches are highly preferred for PPI prediction [17]. The computational methods utilize protein-wide PPIs and provide deep insights into the biological processes [18]. Although various experimental techniques are available to detect and validate PPIs, such techniques are time-consuming and labor-intensive [19]. The current study is the first to delineate the PPIs between *Triticum aestivum* and *Tilletia* species using two computational approaches—interolog and domain-based methods—on the genome-wide scale followed by a detailed functional enrichment, network visualization, and identification of protein hubs, transcription factors, etc.

## 2. Results and Discussion

The genome-wide protein–protein interactions between *T. aestivum* and *Tilletia* species were predicted using two computational approaches: the interolog and domain-based approaches. We employed 112 randomly generated BLAST parameter combinations based on sequence identity (30%, 40%, 50%, and 60%), e-value (1 × 10^−4^, 1 × 10^−5^, 1 × 10^−10^, 1 × 10^−20^, 1 × 10^−25^, 1 × 10^−30^, and 1 × 10^−50^) and sequence coverage (30%, 40%, 50%, 60%, and 80%). The BLAST combinations and the corresponding interactions found for both the fungal species are presented in Supplementary Material File S1 (Excel Sheets S2 and S3).

For *T. aestivum*–*T. caries*, the interactome consisted of 46,557,278 interactions (involving 83,948 host and 4612 pathogen proteins) combined from both the computational approaches, while in *T. aestivum*–*T. laevis*, a smaller number of interactions were predicted in the interactome with 44,725,235 interactions (83,942 host and 4380 pathogen proteins). The predicted interactions are available in Supplementary Material File S2 (Excel Sheets S1–S6). Further, the effector proteins and secretory proteins predicted using EffectorP and SignalP, respectively, were collectively identified in the predictions, and referred to as “effectors” throughout the research analysis. In the predicted interactions, 648 *T. caries* effectors and 575 *T. laevis* effectors were identified. Therefore, we selected the combination with sequence identity ≥ 30%, e-value ≤ 1 × 10^-4^, and coverage ≥ 40%, based on the maximum number of effectors predicted in this combination as compared to other combinations. The detailed predicted PPIs from *T. aestivum*–*T. caries*, and *T. aestivum*–*T. laevis* interactomes are described in Table 1 and Table 2, respectively.

### 2.1. HPIs from the Common Subnetwork (T. aestivum vs. T. caries/T. laevis)

As discussed in the Materials and Methods section, using the ortholog approach, we obtained 423 proteins from both the *Tilletia* species that are orthologs of each other. Subsequently, we identified 79,758 host proteins interacting with these fungal protein orthologs. These proteins were involved in 5,075,732 interactions (referred to as the common subnetwork), which were then taken into consideration for various functional and network analyses.

#### 2.1.1. Protein Hubs Reveal the Major Proteins Involved in the Infection Mechanism

The simultaneous physical interactions between proteins are responsible for the accomplishment of their functions. Therefore, the knowledge of interaction mechanisms is crucial to better understand biological processes [20]. Biological networks enable us to identify the important hubs, and help us to understand their role in various biological processes and molecular mechanisms [21]. In addition, the identification of such interactions is likely to give an indication of the functions of those proteins with unknown functions. To determine the major hubs from the common subnetwork, the node degree was taken into consideration, which is briefly described below.

#### 2.1.2. Degree

On an average, the degree of *T. aestivum* and *Tilletia* proteins (orthologs) was found to be 64 and 11999, respectively (Supplementary Material File S3, Excel Sheets S1 and S2). As expected, the degree of pathogen proteins was observed to be significantly higher than that of the host proteins in the common subnetwork. In the study, a large number of protein hubs were identified from the common subnetwork, of which the top 20 hubs from each species have been discussed below.

#### 2.1.3. *Tilletia* Hubs

In the common subnetwork, the pathogen hubs (Supplementary Material, Figure S1A) showed that a ubiquitin-like domain formed the largest hub, involving *Tilletia* proteins OAJ10932/KAE8196026.1 and OAJ25570/KAE8207011.1. A previous study demonstrated the role of ubiquitin-like activating enzymes, BcAtg3 (E2) and BcAtg7 (E1), in the fungus *Botrytis cinerea*. A yeast two-hybrid system confirmed the physical interaction of both the enzymes. Further, the involvement of *BcATG3* and *BcATG7* was found to be essential in the process of autophagy, sclerotial formation, and virulence of *B. cinerea* [22]. Another cluster consisting of OAJ21901/KAE8193768.1, OAJ25001/KAE8207462.1, OAJ08159/KAE8180887.1, OAJ23224/KAE8204607.1, OAJ26348/KAE8189728.1, OAJ27126/KAE8208298.1, OAJ27114/KAE8206812.1, OAJ25550/KAE8196914.1, and OAI98851/KAE8183999.1 proteins was found to participate in the small GTPase domain and the small GTP-binding protein domain. Small GTPases, such as Rho, Ran, and Ras, are well characterized and known for the regulation of fungal virulence and the production of ROS species, which are essential biological features of fungal colonization in plants [23]. qRT-PCR analysis of *PsRan*, a Ran gene from *Puccinia striiformis* f. sp. t*ritici* (*Pst*), revealed increased transcript levels of *PsRan* in wheat leaves after inoculation with *Pst*. The study also showed the involvement of *PsRan* in fungal growth and development [24]. Rho3, MoMsb2, Erl, and Ras2 play important roles in the appressoria formation and virulence of *Magnaporthe oryzae* [25,26]. A cluster of pathogen proteins, OAJ23752/KAE8187483.1, OAJ10309/KAE8196172.1, and OAJ11697/KAE8188801.1, is involved in a ubiquitin-conjugating enzyme family. The protein hub “OAJ26088/KAE8189542.1” plays a role in the small ubiquitin-related modifier (SUMO)-conjugating enzyme Ubc9. A study on the rice blast fungus *Magnaporthe oryzae* suggested that SUMO-conjugating enzymes are important for the process of SUMOylation, which regulates fungal growth and pathogenicity [27]. The protein hub OAJ25070/KAE8192066.1 was found to belong to the AAA-ATPase domain. Another hub of pathogen proteins (OAJ15999/KAE8183760.1, OAJ15730/KAE8206910.1) was involved in the thioredoxin domain. A study on *Fusarium graminearum* showed that FgTRR, an ortholog of thioredoxin reductase (TRR), is located in the cytoplasm and involved in the virulence of the pathogen [28]. Two proteins, OAJ24420/KAE8208796.1 and OAJ25043/KAE8192521.1, belonged to the RNA recognition motif/RNA-binding domain. In *Verticillium dahliae*, the RNA-binding protein (VdNop12) was found to be crucial for cold adaptation and causing pathogenesis in cotton plants [29]. Similarly, RNA-binding proteins were found to play a role in pathogenic development in *Ustilago maydis* [30].

#### 2.1.4. *Triticum aestivum* Hubs

The analysis of protein hubs revealed that all the host protein hubs (Supplementary Material, Figure S1B) were found to be protein kinases. The most interconnected host hubs (TraesCS4D02G250600.1.cds1, TraesCS7A02G437400.1, TraesCS3A02G306000.1, TraesCS5A02G255500.1.cds1, TraesCS5D02G263800.1.cds1, TraesCS5B02G254600.1.cds1, TraesCS4B02G260700.1.cds1, TraesCS2D02G120200.1, and TraesCS2A02G577400.1) belonged to the serine/threonine (Ser/Thr) protein kinase family. Ser/Thr protein kinases play an essential role in the plant stress response by regulating different signaling pathways. SnRK2, a subfamily of SnRK Ser/Thr protein kinases, is involved in the ABA signal transduction pathway during salt stress [31]. In tomato, a Ser/Thr kinase-encoding gene (*Pto*) was reported to confer resistance against bacterial speck disease. Pto was further found to specifically phosphorylate Pto-interacting 1 (Pti1), and the expression of the *Pti1* transgene resulted in an accelerated hypersensitive response in tobacco against *Pseudomonas syringae* pv. t*abici* expressing *avrPto* [32]. The host hub “TraesCS6D02G339600.1”, interacting with 290 pathogen proteins, belonged to the heat shock protein (Hsp) 70 family. Heat shock proteins are considered as critical in the plant’s response to various environmental stresses and disease resistance. In tobacco, Hsp70 proteins were suggested to be involved in the unfolded protein response (UPR) during biotic and abiotic stresses [33]. A study revealed that the isoforms of Hsp70 and Hsc70 transcripts were found to be highly induced during infection with *Nicotiana benthamiana* that causes cucumber necrosis virus [34]. The protein hub “TraesCS6B02G232400.1” contains the mitogen-activated protein kinase kinase kinase (MAPKKK) NPK1. The MAPK pathway comprises three different protein kinases (MAPKKK, MAPKK, and MAPK) involved in phosphorylation reactions, resulting in the induction of immune responses in the cell [35]. In Arabidopsis, MAPKKK-dependent MAPK cascades (MEKK-1-MKK1/MKK2-MPK4) are reported to be positive regulators of the innate immune responses against the necrotrophic fungus *Botrytis cinerea* and the bacteria *P. syringae* pv. tomato DC3000 [36]. In another study, the constitutive expression of tobacco MAPKKK (NPK1) in transgenic maize showed increased drought tolerance [37]. Further, the NPK1 gene, a MAPKKK ANP orthologue, was shown to be actively involved in multiple stress conditions in plants [38]. Seven protein hubs (TraesCS7A02G352200.1, TraesCS3D02G295900.1, TraesCS6B02G162900.1.cds1, TraesCS3B02G330500.1, TraesCS4B02G168200.1.cds1, TraesCS3A02G135700.1.cds1, and TraesCS4D02G179700.2) were annotated as CBL-interacting protein kinases (CIPKs), which are known to regulate numerous cell signals and enhance stress tolerance in plants [39]. Researchers have previously determined the role of *TaCIPK10*, a CIPK homologue gene, in wheat resistance to *Puccinia striiformis* f. sp. t*ritici*. *TaCIPK10* was immediately induced upon infection with *Puccinia striiformis* and salicylic acid treatment. Further, TaCIPK10 was found to positively regulate wheat resistance against *Puccinia striiformis* by transmitting Ca^2+^ signals [40]. Moreover, the analysis revealed that the host hubs TraesCS2D02G104200.1 and TraesCS2A02G104500.1 belonged to the AGC protein kinase family. Although little information is available on the function of AGC kinases, they are suggested to function in the regulation of growth and the plant defense response against various pathogens. *TaAGC1*, a wheat AGC kinase gene, was found to be a positive regulator against infection with the fungus *Rhizoctonia cerealis*. *TaAGC1* showed enhanced expression in the resistant wheat line CI12633, while reduced expression was observed in the susceptible line Wenmai 6 [41].

**Figure 1:**
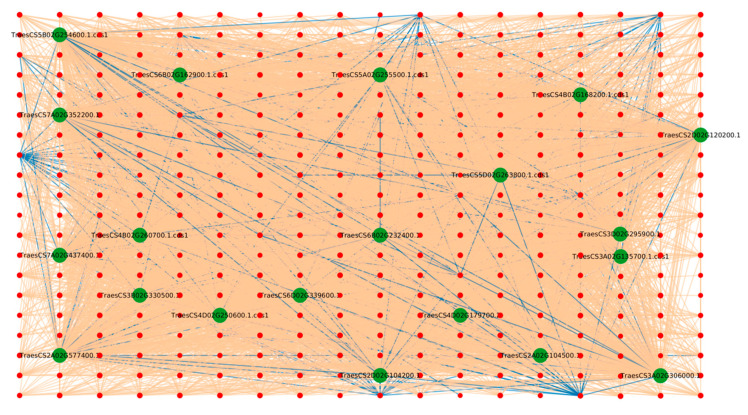
Protein–protein interaction network for the top 20 host protein hubs. Green nodes represent host proteins and red nodes are pathogen proteins. Orange edges depict the interactions from the interolog-based approach, while blue edges belong to the domain-based approach.

The hub network (Figure 1) analysis revealed that, based on degree, the top 20 pathogen proteins are involved in interaction with the top 20 host proteins, suggesting that the pathogens primarily hijack the protein kinase machinery in the host, thus controlling various processes of the plant immune system. The host protein hubs play a role in plant defense, while the pathogen proteins are involved in the processes that enhance fungal development and pathogenicity. Studying the interactions between the potential host and pathogen proteins can further help understand the infection mechanism, and how the pathogens evolve inside the host.

### 2.2. GO Enrichment Analysis of the Proteins Involved in the Interactions

The GO enrichment analysis of the host and pathogen proteins involved in the common subnetwork was performed using the enrichment score [-log10(*p*-value)]. The enrichment analysis of pathogen proteins revealed that 423 pathogen proteins were enriched in 578 GO terms representing the three GO categories (biological process, cellular component, and molecular function) (Supplementary Material, Figure S2). In the molecular function category, the highly enriched GO terms were GTPase activity (GO:0003924), peptidase activity (GO:0008233), and hydrolase activity (GO:0004553). The biological process category comprises proteolysis (GO:0006508), carbohydrate metabolic process (GO:0005975), and protein peptidyl-prolyl isomerization (GO:0000413). Apart from this, the over-represented GO terms in the cellular component category involved endoplasmic reticulum (GO:0005783), ribosome (GO:0005840), and respirasome (GO:0070469). The over-represented GO terms suggest the role of pathogen proteins in increased pathogen virulence, colonization, and the suppression of the plant defense mechanism, which has previously been validated in the literature [42,43]. The detailed GO enrichment of *Tilletia* species proteins is available in Supplementary Material File S3 (Excel Sheet S3), along with other important parameters, such as protein count and adjusted *p*-value.

**Figure 2:**
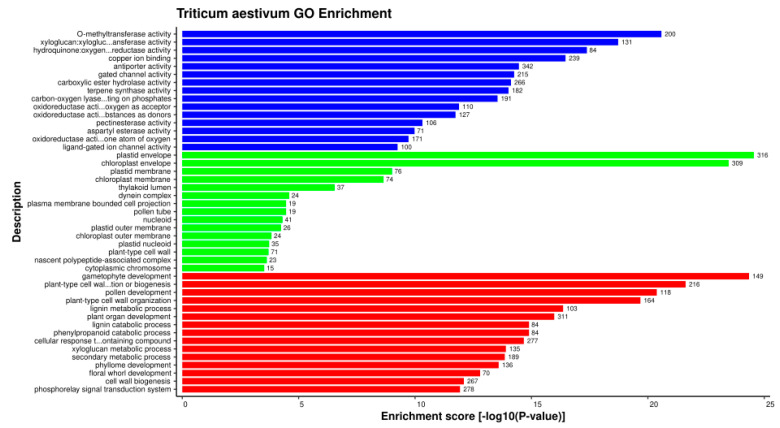
Over-representation of the top 15 GO terms (from each GO category) of host proteins based on enrichment score showing molecular function (blue), cellular component (green), and biological process (red).

On the other hand, 79,758 common wheat proteins involved in interactions with the pathogen proteins in the common subnetwork were enriched in 3562 GO terms (Supplementary Material File S3, Excel Sheet S4). The top 15 GO terms from each GO category are represented in Figure 2. The most enriched GO terms in the biological process category involved gametophyte development (GO:0048229), pollen development (GO:0009555), and plant-type cell wall organization or biogenesis (GO:0071669). Ubiquitination, a process involved in various plant defense signaling pathways [44], requires three ubiquitin-conjugating enzymes, viz. E1, E2, and E3, to form the isopeptide bond. A study on Arabidopsis revealed the involvement of UBC22, belonging to the E2 subfamily, in female gametophyte development [45]. *Osg1*, a β-1,3-glucanase-encoding gene, showed its expression during the early microspore and middle microspore stages in the floret. Upon silencing the expression of *Osg1*, male sterility was observed in transgenic rice, thus showing its role in male gametophyte development. β-1,3-glucanases play a significant role in defending the plant by degrading the cell wall of fungal pathogens [46]. In wheat, a higher expression of β-1,3-glucanase was observed in the resistant genotype in response to spot blotch disease as compared to the susceptible genotype [47].

In the cellular component category, the proteins were found to be enriched in the plastid envelope (GO:0009526), chloroplast envelope (GO:0009941), and plastid membrane (GO:0042170). Hydroperoxide lyase (HPL), localized in outer envelope of the plastid, catalyzes C6-aldehydes, which are explicitly involved in pathogen defense [48,49]. In Arabidopsis, studies report that upregulated AtHPL expression was observed during fungal attack by *Botrytis cinerea*, which further led to increased levels of C6-aldehydes at the site of pathogen penetration, thus inhibiting the growth of the pathogen [50]. The inner envelope membrane of the chloroplast serves as the site for the synthesis of α-tocopherol and plastoquinone-9 [51], which are reported to be involved in plant defense mechanisms. α-tocopherols are essential antioxidants that prevent disease in plants by functioning in various processes, such as intracellular signaling and the activation of defense responses in stress conditions [52]. *Mesembryanthemum crystallinum* plants, when treated with *Botrytis cinerea*, showed that plastoquinone regulates plant biotic stress in the redox state, and an accelerated hypersensitive-like response was observed in the reduced state of plastoquinone [53].

**Figure 3:**
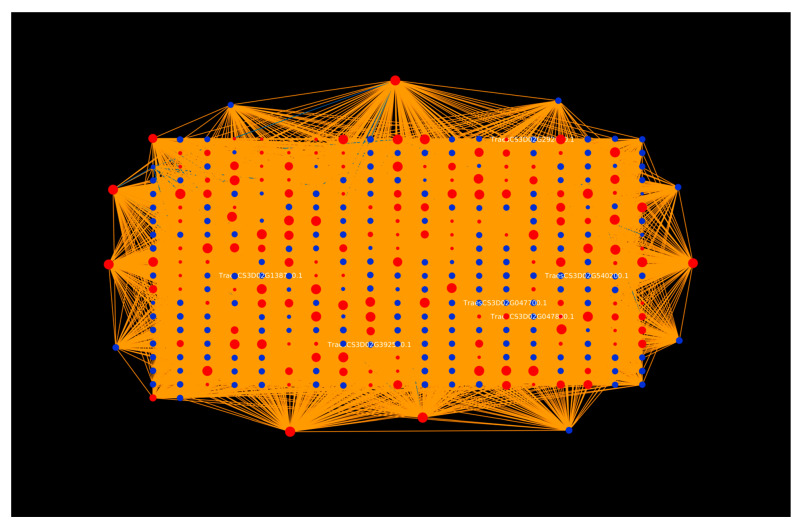
Visualization of top GO term (GO:0008171, O-methyltransferase activity) from the molecular function category. Blue nodes represent host proteins, red nodes are pathogen proteins. Orange edges depict the interactions from the interolog-based approach, while blue edges belong to the domain-based approach.

In the molecular function category, O-methyltransferase activity (GO:0008171), xyloglucan:xyloglucosyl transferase activity (GO:0016762), and hydroquinone:oxygen oxidoreductase activity (GO:0052716) were found to be abundant. Various plant secondary metabolites, such as alkaloids, flavonoids, and phenylpropanoids, catalyzed by S-adenosyl-L-methionine-dependent O-methyltransferases (OMTs), play critical roles in plant growth and development [54]. An important class of OMTs, caffeic acid 3-O-methyltransferases (COMTs; EC 2.1.1.6), are involved in lignin biosynthesis. At the time of plant–pathogen interaction, lignin deposition in the cell wall simultaneously acts as a physical barrier against pathogens, increases the host’s resistance to toxins, and restricts the diffusion of nutrients from the host to the pathogens [55]. When inoculated with the necrotrophic fungus *Rhizoctonia cerealis*, a wheat COMT gene, *TaCOMT-3D* (localized in chromosome 3D), showed increased levels of expression. The silencing of *TaCOMT-3D* showed a high susceptibility to sharp eyespot disease, while the overexpression of *TaCOMT-3D* enhanced the resistance of transgenic wheat lines against the disease. The overexpression of *TaCOMT-3D* also led to an increase in lignin accumulation [56]. In our study, we identified six proteins (Figure 3) belonging to *T. aestivum* chromosome 3D (TraesCS3D02G392500.1, TraesCS3D02G540200.1, TraesCS3D02G047700.1, TraesCS3D02G047800.1, TraesCS3D02G138700.1, and TraesCS3D02G292000.1), that are involved in O-methyltransferase activity, thus providing strong evidence for their role in the plant defense against fungal disease. A study on *Arabidopsis* quinone reductases (QRs), *Nqr* and *Fqr*, revealed an altered interaction between QR knockout (KO) lines and the necrotrophic fungi *Botrytis cinerea* and *Sclerotinia sclerotium*, whereby the KO lines (nqr^-^ and fqr1^-^) were found to be less susceptible to the fungi when compared to the wild-type lines, while the overexpression line FQR1^+^ showed an increased hypersensitive response against the pathogens [57].

**Figure 4:**
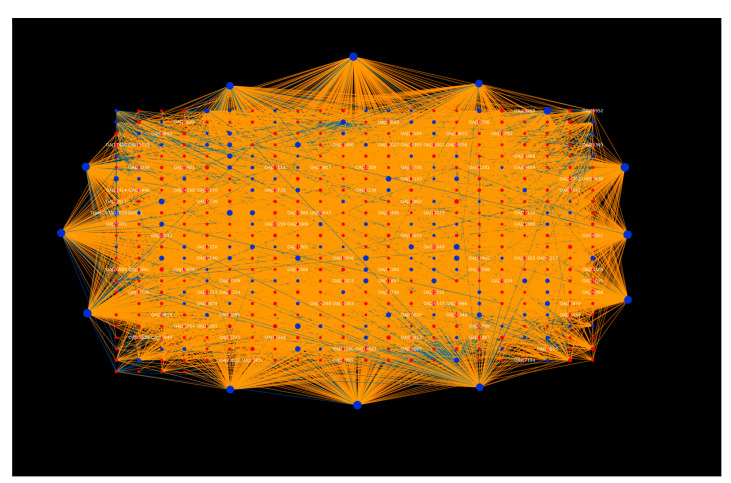
Visualization of the top GO term (GO:0048229, gametophyte development) from the biological process category. Blue nodes represent host proteins, red nodes are pathogen proteins. Orange edges depict the interactions from the interolog-based approach, while blue edges belong to the domain-based approach.

**Figure 5:**
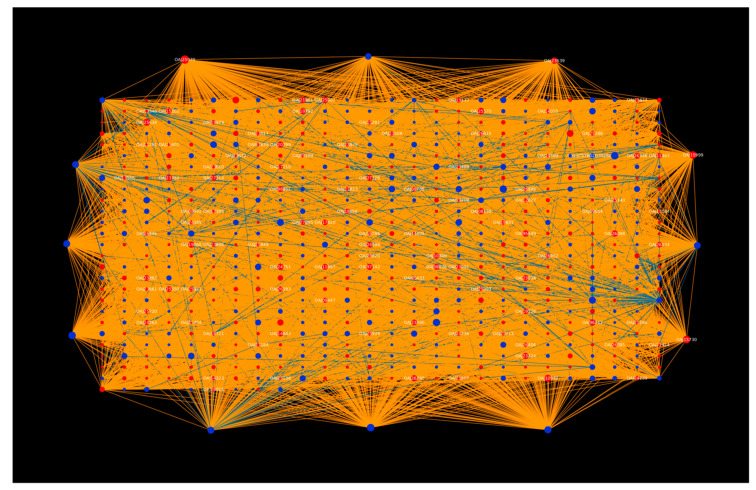
Visualization of the top GO term (GO:0009526, plastid envelope) from the cellular component category. Blue nodes represent host proteins, red nodes are pathogen proteins. Orange edges depict the interactions from the interolog-based approach, while blue edges belong to the domain-based approach.

Further, the network analysis of the top GO terms for each category revealed that the host protein “TraesCS7B02G392600.1” was found to be enriched both in gametophyte development (GO:0048229) (Figure 4) and the plastid envelope (GO:0009526) (Figure 5), interacting with 105 pathogen proteins. The TraesCS7B02G392600.1 protein was also found to be involved in other GO terms, including response to osmotic stress (GO:0006970), response to salt stress (GO:0009651), ATP biosynthetic process (GO:0006754), plant-type vacuole (GO:0000325), proton-transporting V-type ATPase (GO:0033180), ion channel activity (GO:0005216), and enzyme binding (GO:0019899). Additionally, this protein also showed involvement in oxidative phosphorylation (ko00190) and the mTOR signaling pathway (ko04150). The results imply that this protein is highly involved in plant defense mechanisms and plays a role in various biological processes during stress conditions. In addition, 105 pathogen proteins interacting with the above-mentioned host were involved in GTPase activity (GO:0003924), exopeptidase activity (GO:0008238), proteolysis (GO:0046034), small GTPase mediated signal transduction (GO:0007264), and the inner mitochondrial membrane protein complex (GO:0098800). These interactions can be considered as potential candidates for experimental analysis, thus providing insights into the infection mechanism and other biological processes pertaining to the disease.

### 2.3. Analysis of Over-Represented KEGG Pathways

KEGG enrichment provides in-depth understanding of the proteins, which play a role in different biological pathways. In line with this, KEGG enrichment analysis of the host and pathogen proteins involved in the common subnetwork was performed. The pathogen proteins were found to be enriched in 257 KEGG pathways (Supplementary Material File S3, Excel Sheet S5) involving plant hormone signal transduction (ko04075), MAPK signaling pathway-plant (ko04016), protein processing in endoplasmic reticulum (ko04141), and NOD-like receptor signaling pathway (ko04621). The top 20 enriched pathways for pathogen proteins are shown in Supplementary Material, Figure S3.

**Figure 6:**
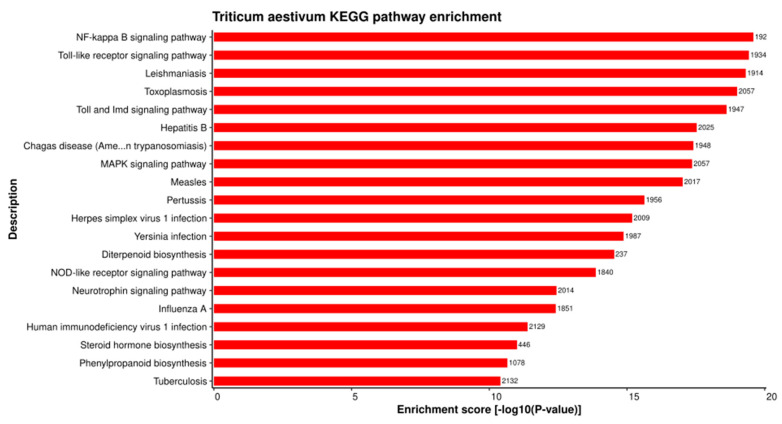
KEGG pathway enrichment analysis of the host proteins involved in HPIs. Top 20 pathways are represented on the basis of enrichment score.

**Figure 7:**
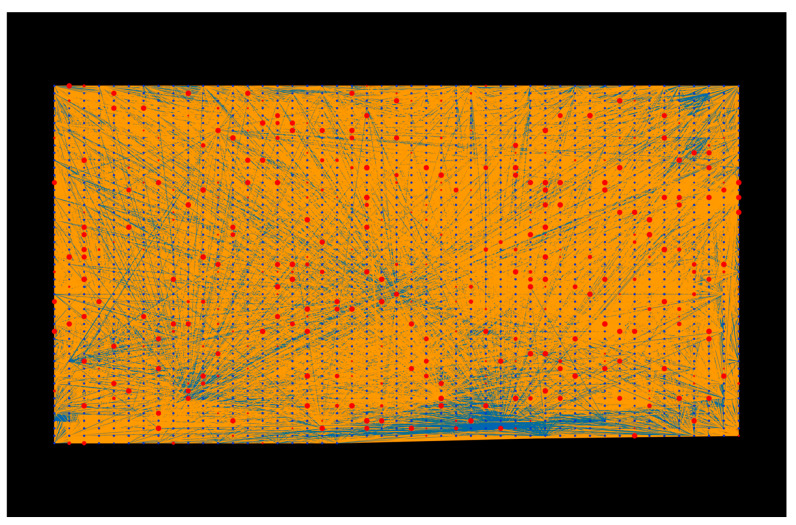
Visualization of the top KEGG pathway (ko04064, NF-kappa B signaling). Blue nodes represent host proteins, red nodes are pathogen proteins. Orange edges depict the interactions from the interolog-based approach, while blue edges belong to the domain-based approach.

KEGG pathway enrichment analysis demonstrated that the host proteins interacting with the *Tilletia* proteins were over-represented in 399 pathways (Supplementary Material File S3, Excel Sheet S6), of which the top 20 pathways are represented in Figure 6. The highly enriched pathways included the NF-kappa B signaling pathway (ko04064) (Figure 7), Toll-like receptor signaling pathway (ko04620), and the MAPK signaling pathway-plant (ko04016). The transcription factor nuclear factor kappa B (NF-κB) is reported to regulate immune responses against various extracellular stimuli (such as pathogens) or intracellular stress signals [58]. A study on Arabidopsis *NIM1* gene mutants suggested the interaction of the NIM1 protein with NF-κB-related transcription factor, which helps in the induction of systemic acquired resistance (SAR) gene expression and also triggers gene-for-gene resistance against the disease [59]. Further, the Toll-like receptors (TLRs) activate the genes that are responsible for the initiation of immune responses in plants [60]. Mitogen-activated protein kinases (MAPKs), a class of conserved protein kinases, are known to play a critical role in signaling mechanisms during various environmental stresses by associating extracellular stimuli with intracellular responses [61]. In rice, MAPKs have been reported to show resistance against leaf blight disease, caused by *Xanthomonas oryzae*. The overexpression of rice group C MAPK (OsMPK7) and its upstream MAPK kinase (OsMKK3) in leaves and roots showed inhibition against *X. oryzae* infection, while the silencing of OsMPK7 accounted for disease susceptibility [62]. The enrichment analysis results indicate that the host proteins are involved in the pathways related to the generation of defense-related signals against various stresses, while the pathogen proteins are associated with the pathways that regulate the pathogenesis and metabolism of fungal proteins inside the host cell. Both the host and pathogen proteins were found to be enriched in the MAPK signaling pathway, suggesting a potential crosstalk between the host and pathogen.

### 2.4. The Majority of Host–Pathogen Interactions Were Localized in the Plastid of Host Cells

Living cells involve a complex machinery that sorts and sends newly synthesized proteins into final locations (compartments) in the cell [63]. The subcellular localization of proteins is a basic part in the quest of determining the function of proteins within the cellular compartment. Protein subcellular localization also gives an inference about the pathway to which an enzyme belongs [64].

**Figure 8:**
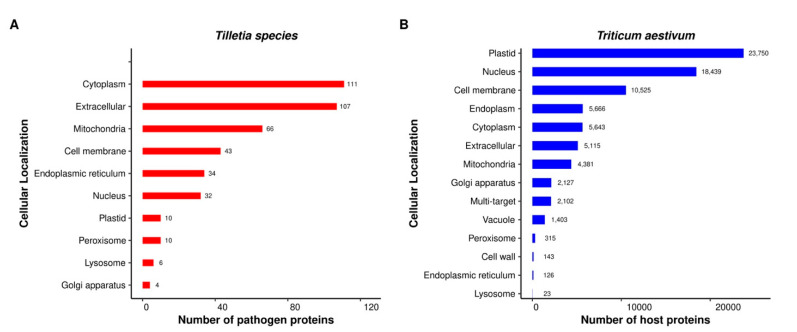
Subcellular localization of the pathogen proteins (**A**) and host proteins (**B**) involved in the common subnetwork.

In our analysis, the subcellular location of *T. aestivum* and *Tilletia* species proteins was predicted to gain better insights into the location of host–pathogen interactions. We found that the majority of the pathogen proteins in the common subnetwork were either extracellular (25.29%) or localized in the cytoplasm (26.24%) and mitochondria (15.6%), followed by the cell membrane (10.16%), endoplasmic reticulum (8.04%), nucleus (7.56%), plastid (2.4%), peroxisome (2.4%), lysosome (1.41%), and Golgi apparatus (0.94%) (Figure 8A). The subcellular localization of ubiquitin-like activating enzymes (BcAtg3 and BcAtg7) in *Botrytis cinerea* was determined using fluorescence assays, which revealed that both the enzymes were localized in the cytoplasm [22]. In *Saccharomyces cerevisiae*, the pathogenesis-related thioredoxin reductases were found to be localized in the cytoplasm and mitochondria [65,66].

On the other hand, the host proteins associated with fungal proteins in the interactions were mostly located in the plastid (29.77%), nucleus (23.12%), and cell membrane (13.19%) (Figure 8B). In rice, the OsVQ domain proteins were shown to play an important role against various biotic and abiotic stresses. The study postulated that the OsVQ proteins were localized in the nucleus and plastid [67], which is in line with our subcellular localization prediction. A total of 2102 host proteins were found to be localized at multiple sites, implying the role of host proteins in various processes at different locations inside the cell. Such multifunctional proteins are also known as moonlighting proteins [68]. The detailed subcellular localizations of the predicted host and pathogen proteins are available in Supplementary Material File S3 (Excel Sheets S7: Pathogen, and Excel Sheets SS8: Host). Our analysis further suggested that the pathogen proteins targeted host proteins mainly in the plastid (Supplementary Material File S3, Excel Sheet S9).

### 2.5. Unique Interactions between Host and Pathogens

In this study, we were interested in further comparing the species-specific HPIs and the functional differences between *T. caries* and *T. laevis*. In line with this, we identified 225 unique *T. caries* proteins interacting with 78,294 host proteins, accounting for 2,514,109 interactions (Supplementary Material File S4, Excel Sheets S1–S3). On the other hand, 152 unique *T. laevis* proteins were identified that were involved in 1,865,266 interactions with 76,903 host proteins (Supplementary Material File S4, Excel Sheets S4–S6). The comparison between the predicted HPIs suggests that the number of interactions with the host proteins in the case of *T. laevis* were much less than those of *T. caries*. Thus, it can be inferred that infection can occur even with a smaller number of interactions between the host and pathogen proteins. Further, we compared the enrichment analysis results of the unique proteins with the common subnetwork.

#### 2.5.1. Functional Analysis of Unique *T. caries* Proteins in the Predicted PPIs

The functional enrichment analysis indicated that most of the over-represented GO terms were similar between the common subnetwork and *T. caries*. These included the lipid catabolic process (GO:0016042), proteolysis (GO:0006508), triglyceride lipase activity (GO:0004806), GTPase activity (GO:0003924), nucleosome (GO:0000786), and DNA packaging complex (GO:0044815). However, in comparison to the common subnetwork, we also found 70 GO terms that were unique to *T. caries,* comprising nucleobase-containing compound kinase activity (GO:0019205), phosphotransferase activity (GO:0016776), the regulation of phosphate metabolic process (GO:0019220), and small molecule catabolic process (GO:0044282). For unique KEGG pathways in *T. caries*, ABC transporters (ko02010) and arginine and proline metabolism (ko00330) were found to be over-represented. The GO and KEGG enrichment analysis for *T. caries* proteins is detailed in Supplementary Material File S5 (Excel Sheets S1 and S2). Various studies in the past have shown that these GO terms and KEGG pathways aid in pathogen survival and virulence [69,70,71,72].

On the other hand, for the host proteins associated with the unique *T. caries* proteins, only two GO terms were found to be unique, viz. GO:0032367 (intracellular cholesterol transport) and GO:0030301 (cholesterol transport), which are related to humans and do not play a role in the plant defense system. All the enriched KEGG pathways were found to be similar for the host proteins associated with the unique *T. caries* proteins and the common subnetwork.

#### 2.5.2. Functional Analysis of Unique *T. laevis* Proteins in the Predicted PPIs

The GO enrichment analysis of unique *T. laevis* proteins revealed analogous patterns, similar to the unique *T. caries* proteins and the common subnetwork. In comparison with the common subnetwork, the unique GO terms associated with *T. laevis* involved the cellular carbohydrate metabolic process (GO:0044262), regulation of kinase activity (GO:0043549), NAD binding (GO:0051287), oxidoreductase activity (GO:0016627), and cytosol (GO:0005829). We also found a few KEGG pathways unique to *T. laevis*, for example, isoquinoline alkaloid biosynthesis (ko00950) and arginine and proline metabolism (ko00330). The enrichment analysis for unique *T. laevis* proteins is available in Supplementary Material File S5 (Excel Sheets S3 and S4).

On the other hand, it was observed that the host proteins associated with the unique *T. laevis* proteins were enriched in similar GO terms. The GO term “condensed chromosome kinetochore” (GO:0000777) was found to be unique to *T. laevis* host proteins, and is irrelevant to plants. No KEGG pathway was retrieved that was unique to the host proteins of *T. laevis*. In addition, the subcellular localization analysis indicated no unique patterns for the localization of the proteins.

### 2.6. Novel Host Targets Show Resistance to Common Bunt Disease

The innate immune system in plants deploys various specialized genes, known as R (resistance) genes, responsible for the detection of pathogens and the initiation of specific immune responses. The recent surge in genomic resources, combined with the advancement in plant breeding programs, has accelerated the identification and cloning of resistance genes in wheat [73]. Due to the complex nature of the hexaploid wheat genome, the study of R gene evolution provides deeper insights into the immune mechanisms, resulting in novel candidates for disease resistance.

Researchers in the past have identified common bunt resistance QTLs/SNPs located on different chromosomes in wheat using various genomic techniques, such as genotyping-by-sequencing (GBS), genome-wide association study (GWAS), and marker-assisted selection (MAS). A genome-wide association study in wheat identified 15 common bunt resistance-related SNPs on chromosomes 1B, 2A, 2B, 3D, 4A, 7A, and 7B, of which five potential SNPs were located on chromosomes 2A, 3D, and 4A [74]. In another paper, GWAS found 123 SNPs on 14 chromosomes (1A, 1B, 2B, 3A, 3B, 4A, 5A, 5B, 5D, 6A, 6B, 7A, 7B, and 7D) that were associated with the Nebraska race of common bunt resistance. Among those identified, a significant number of SNPs were located on chromosomes 1B (31 SNPs) and 6B (28 SNPs). Some of the identified SNPs on chromosomes 1B and 2B were also found to be associated with bunt resistance genes (*Bt1*, *Bt4*, *Bt5*, *Bt6*, *Bt12*) [75]. Common bunt resistance was also found to be regulated by the QTLs *QBt.ifa-1BS*, *QBt.ifa-1AL*, and *QBt.ifa-7AL* that were mapped to chromosomes 1A, 1B, and 7A, respectively. Several major and minor QTLs on chromosome 1B have been mapped, showing that chromosome 1B is highly involved in disease resistance [76]. With the use of genomics and bioinformatics, a wheat R-gene atlas was constructed consisting of resistance genes against wheat pathogens. The R-gene atlas contains 16 genes (*Bt1*-*Bt15*, *Btp*) associated with bunt, and these genes were located on chromosomes 1B, 2B, 2D, 3B, and 6D [77]. Combining the information from the above-mentioned resources, we identified the proteins belonging to the bunt resistance chromosomes. The analysis resulted in 69,215 host proteins that can serve as novel targets to understand the immune responses implicated by the host proteins during stress conditions. These host proteins were involved in 4,411,628 interactions with 423 pathogen proteins, which can be further considered as potential candidates for further studying the disease mechanism. The maximum number of interactions belonged to chromosome 2B, followed by chromosomes 2D and 2A. The interactions associated with bunt resistance chromosomes are available in Supplementary Material File S6 (Excel Sheets S1–S18).

### 2.7. Identification of Stress-Related Transcription Factors in T. aestivum

Transcription factors (TFs) are proteins that critically regulate gene expression upon perceiving stress-related signals and further activate defense-related pathways, such as the ABA signaling pathway, salicylic acid signaling pathway, and others [78]. In our analysis, we identified 5455 wheat proteins that served as transcription factors (Supplementary Material File S7, Excel Sheet S1), controlling various defense response signals during biotic and abiotic stresses. The identified proteins were classified into 28 TF families that were highly responsive during environmental cues. These host proteins were found to interact with 334 pathogen proteins, accounting for 322,816 PPIs (Supplementary Material File S7, Excel Sheet S2). According to previous reports, the transcription factor families such as bHLH (basic helix-loop-helix), NAC (NAM, ATAF1/2, and CUC2), MYB (myeloblastosis related), bZIP (basic leucine zipper), ERF (ethylene responsive factor), WRKY, AP2 (APETALA2), and CAMTA (CaM-binding transcription activator) are considered to play a critical role during the defense response against pathogens [79,80].

## 3. Materials and Methods

**Figure 9:**
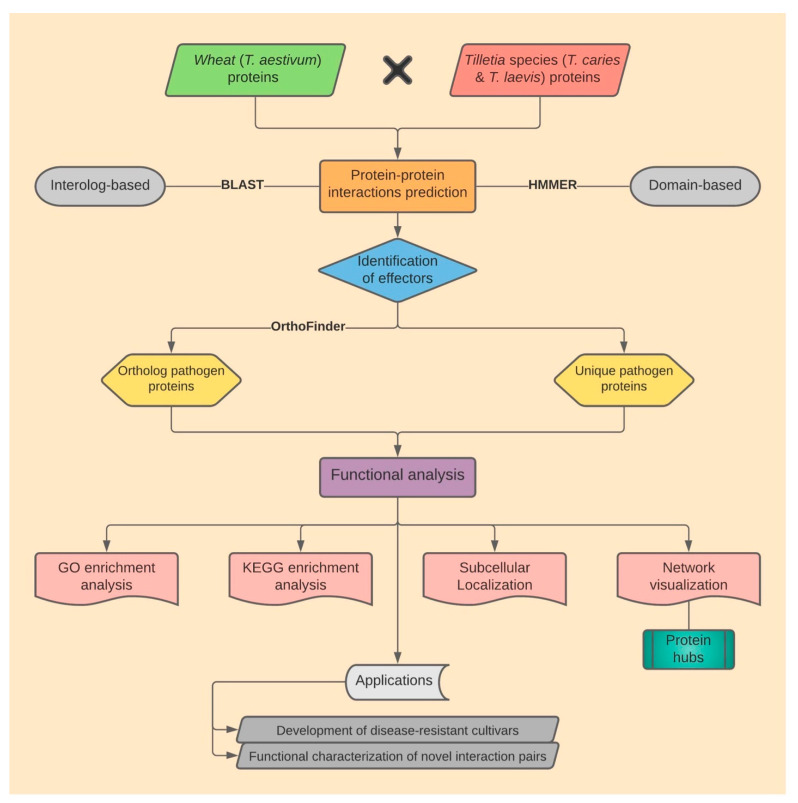
Overall workflow for protein–protein interaction prediction in the wheat–common bunt (*T. aestivum*–*Tilletia* sp.) system.

An overview of the workflow we developed for deciphering the wheat–common bunt PPIs is presented in Figure 9. The detailed steps are described below.

### 3.1. Datasets

The proteomes of *Triticum aestivum* and the two *Tilletia* species were collected from different sources: *T. aestivum* (133,346 proteins) from Ensembl Plants (http://plants.ensembl.org/index.html (accessed on 20 April 2021)), *T. caries* strain DAOM 238032 (10,204 proteins) from Ensembl Fungi (https://fungi.ensembl.org/index.html (accessed on 20 April 2021)), and *T. laevis* strain DAOMC 238040 (9651 proteins) from the National Center for Biotechnology Information (NCBI) (https://www.ncbi.nlm.nih.gov/ (accessed on 20 April 2021)). To ensure non-redundancy, the proteomes of the *Tilletia* species were analyzed with CD-HIT [81] at 100% identity, which resulted in 10,170 *T. caries* and 9637 *T. laevis* proteins that were further employed for the prediction of HPIs. Being hexaploid (AABBDD) in nature, a high number of isoforms were found in the wheat proteins. To overcome this complexity, the longest isoforms were considered, followed by the genome-wise segregation of the proteins into genome A, genome B, and genome D proteins. There were 2659 unknown proteins that did not belong to any of the genome; hence, these were treated as a separate category. The proteins (genome-wise) were then analyzed with CD-HIT at 100% identity, which led to 104,701 *T. aestivum* proteins in total. Throughout the research analysis, the protein accessions initiated with “Traes”, “OAJ”, and “KAE” refer to *T. aestivum*, *T. caries*, and *T. laevis* proteins, respectively.

To infer the host–pathogen interactions, two approaches were implemented: an interolog-based and a domain-based approach. In the interolog-based approach, seven interaction databases, namely IntAct [82], MINT (Molecular INTeraction database) [83], HPIDB (Host-Pathogen Interaction Database) [84], DIP (Database of Interacting Proteins) [85], BioGRID (Biological General Repository for Interaction Datasets) [86], STRING (Search Tool for the Retrieval of Interacting Genes/Proteins) [87], and PHI-base (Pathogen-Host Interactions database) [88], were employed in the analysis. Each of the above-mentioned databases were downloaded and executed locally.

In the domain-based approach, three domain–domain interaction (DDI) databases, namely 3did (three-dimensional interacting domains) [89], DOMINE (Database of Protein Domain Interactions) [90], and IDDI (integrated domain–domain interaction) [91], were executed locally. In order to predict the domain–domain interactions, the domains for pathogen and host proteins were fetched from the Pfam v31.0 [92] database and were then queried against the three DDI databases. The detailed information of the databases is available in Supplementary Material File S1 (Excel Sheet S1).

### 3.2. Prediction of PPIs between T. aestivum and Tilletia Species

#### 3.2.1. Interolog-Based Prediction

The interolog method, i.e., homology-based prediction of HPIs, is based on the conserved interactions between two proteins in one species that have interacting homologs in other species, i.e., inter-species predictions [93]. This conserved interaction is known as “Interolog”. The analysis is carried out by performing a sequence alignment of the host and pathogen proteins against the seven respective databases (BioGRID, DIP, IntAct, HPIDB, MINT, STRING, and PHI-base) using BLAST v2.7.1 with default parameters (sequence identity, e-value, and sequence coverage), followed by the filtering of the alignments using an appropriate combination of BLAST parameters. For filtering the BLAST alignments, random combinations of sequence identity, e-value, and sequence coverage were generated, and an optimal combination was selected. Previously, there was no leading evidence reporting the standard cutoff for evaluating the BLAST parameters for the prediction of HPIs. In a study on *Mycobacterium tuberculosis* and *Homo sapiens*, researchers predicted HPIs by employing an e-value of 1e-10 and sequence identity of 30% [94]. Other researchers identified dynamic PPIs in cassava by using the following criteria: e-value ≤ 1 × 10^−10^, percent identity ≥ 60%, and percent coverage ≥ 80% in BLASTp sequence alignments [95].

#### 3.2.2. Domain-Based Prediction

In this method, the domains, being the mediators of the interactions, are exploited to infer the PPIs in single organisms [96], i.e., unlike the interolog approach, this approach can be employed only for intra-species predictions. In order to predict HPIs, an algorithm integrates the protein domains with interactions between different proteins of the same organism, and this interaction is estimated using Bayesian statistics [93]. HMMER v3.3.1 (hmmscan), based on hidden Markov models (HMMs), was employed to delineate the domains for host and pathogen from the Pfam database. These domains were further queried locally in SQL for the prediction of PPIs. The filtering of the hmmscan results for *T. aestivum* was performed using an e-value and coverage of 1 × 10^−23^ and 0.2, respectively, while the filtering of the *Tilletia* species was performed using an e-value of 1 × 10^−17^ and coverage of 0.45. The domain-based prediction of PPIs using hmmscan has been successful in various studies [97,98].

### 3.3. Prediction of Effector and Secretory Proteins

Fungal effectors colonize the plant cell, overcome the plant immune system, and suppress the defense system by targeting specific host proteins [99]. Secretory proteins are short proteins that have signal peptides in their amino terminus, which help in translocating the proteins across the host plasma membrane and ultimately alter the host cell’s physiological processes [100]. In view of this, we predicted the effector and secretory proteins in the *T. caries* and *T. laevis* datasets. To identify the proteins serving as effectors, the proteomes of both the species were analyzed on the EffectorP 2.0 web server (http://effectorp.csiro.au/ (accessed on 23 April 2021)) [99], while the fungal secretory proteins were identified using the SignalP 5.0 tool (http://www.cbs.dtu.dk/services/SignalP/ (accessed on 23 April 2021)) [101].

### 3.4. Functional Enrichment Analysis

In order to determine the function/biological pathways of the proteins involved in the predicted HPIs, a functional annotation of the proteins in the predictions was performed. For functional enrichment, gene ontology (GO) and the Kyoto Encyclopedia of Genes and Genomes (KEGG) analyses were performed using the R package clusterProfiler [102]. For the GO enrichment of the host and pathogen proteins, the three GO annotation ontologies, i.e., biological process, cellular component, and molecular function, were considered using the Benjamini and Hochberg test correction method [103] at a p-value cutoff of ≤0.05. KEGG enrichment for the proteins was also performed using the clusterProfiler package at a *p*-value cutoff of 0.05.

### 3.5. Subcellular Localization of the Predicted Proteins

The various proteins synthesized in an organism are destined to perform a specialized function in a specific subcellular compartment of a cell [104]. Plant pathogens secrete effector proteins into host cells, which target specific cell compartments and further subvert the host mechanism to their advantage [105]. Thus, the determination of the subcellular localization of the host and pathogen proteins involved in the interactions is an essential step of the host–pathogen interaction analysis and provides the information about the location of the interactions between host and pathogen proteins. To predict the subcellular localization of the host proteins, we employed the SVM (support vector machine)-based model of Plant-mSubP tool [106]. The subcellular localization of the fungal proteins was predicted using the standalone version of DeepLoc 1.0, a deep learning-based tool for the prediction of eukaryotic protein localization [107].

### 3.6. Comparison between HPIs of T. caries and T. laevis

Since both the *Tilletia* species are highly similar, we decided to find the functional differences between the two fungal species. By comparing the HPIs, we found the proteins that serve as orthologs between *T. caries* and *T. laevis*. Due to the unavailability of a specific database for the *Tilletia* species, the orthologs were predicted using the standalone tool OrthoFinder [108], which provides a phylogenetic-based inference of orthologs between two species. Further, we identified the host proteins interacting with the orthologs. The common PPIs from both the species obtained using fungal protein orthologs and common host proteins were referred to as the common subnetwork, and the functional analysis was carried out using the common subnetwork. The pathogen proteins that were not predicted as orthologs, named as unique proteins, were analyzed separately to find the differences between the two fungal species.

### 3.7. Network Visualization

The generation of enormous interactome data from computational techniques has led to the need for robust analytical strategies to obtain deeper insights into the function of various elements of large-scale datasets [109]. Since proteins play a major role in various biological processes, the systematic study of the PPI network helps in deciphering the underlying cellular mechanisms in a diseased organism or in identifying novel protein targets. The protein interaction network abstracts the relationship between the function and network structure, depending on the functional and physical association [110]. In this study, the visualization of protein networks was carried out using cytoscape [111], the most widely used tool for network analysis. The networks were enhanced using different styles and yFiles layout algorithms in cytoscape and were further analyzed to determine the major protein hubs.

## 4. Conclusions

Host–pathogen interactions illustrate the mechanisms by which pathogens subvert host cellular processes and initiate an infection. The advancement in computational biology and bioinformatics has enabled researchers to extensively study large datasets at the genome-wide scale. Here, the interactome study between *T. aestivum* and the two *Tilletia* species postulated that protein–protein interactions between the host and pathogen forms the basis of disease infection. Using interolog-based and domain-based approaches, we predicted 5,075,732 common PPIs (from *T. caries* and *T. laevis*) involving 79,758 host and 423 pathogen proteins. From the protein hub analysis, we found that the most interconnected host hub was involved in the activity of serine/threonine protein kinases, MAPKKK, and the Hsp70 family, which have been reported to be implicated in defense signals during biotic stress. The GO and KEGG enrichment analysis of the host proteins showed significantly over-represented terms/pathways that play a role in plant defense against pathogens. These involved the GO terms O-methyltransferase activity (GO:0008171), hydroquinone:oxygen oxidoreductase activity (GO:0052716), plastid envelope (GO:0009526), chloroplast membrane (GO:0031969), regulation of signal transduction (GO:0009966), and the KEGG pathways NF-kappa B signaling (ko04064), plant-pathogen interaction (ko04626), plant hormone signal transduction (ko04075), calcium signaling pathway (ko04020), and others. On the other hand, the enrichment analysis of the pathogen proteins suggested that they were involved in virulence. The subcellular localization of the proteins involved in the HPIs exhibited different cellular sites where the host and pathogen proteins are localized. We also identified novel host proteins that serve as R-genes and transcription factors, which can be considered as potential candidates for understanding the common bunt infection mechanism. Various researchers in the past have identified bunt-resistance SNPs/QTLs on different chromosomes, thus validating our predicted PPIs. Furthermore, the data generated from experimental techniques (such as the yeast two-hybrid system and bimolecular fluorescence complementation) and transcriptomics can be integrated with the predicted PPIs, thus improving the quality of the interactome. We believe that the data generated from the current study will be of immense importance to the research community to gain in-depth knowledge of the host defense responses against pathogen attack.

## Figures and Tables

**Table 1:** Predicted PPIs in *T. aestivum*–*T. caries* interactome.

Interaction Database	Number of Interactions	Number of Host Proteins	Number of Pathogen Proteins
**Interolog-based**			
BioGRID	11,343,237	51,839	3574
DIP	1,334,228	27,027	2404
HPIDB	48,331	6779	247
IntAct	4,768,852	48,915	3320
MINT	1,338,779	23,156	2608
PHI-base	28	7	4
STRING	31,159,410	82,876	2638
**Total (Interolog) (I)**	**37,714,442**	**83,639**	**3872**
**Domain-based**			
3DID	1,221,946	27,342	2612
DOMINE	5,803,329	28,007	2623
IDDI	12,112,523	33,619	3235
**Total (Domain) (II)**	**14,307,366**	**35,526**	**3396**
I and II (combined)	46,557,278	83,947	4612
I and II (consensus)	5,464,530	30,629	2401
Interolog (unique)	32,249,912	83,637	3867
Domain (unique)	8,842,836	34,390	3348

**Total (Interolog) (I):** The predicted HPIs from all seven interolog databases were merged and duplicates were removed. **Total (Domain) (II):** The predicted HPIs from all three domain databases were merged and duplicates were removed. **I and II (combined):** The predicted HPIs from both the methods were merged and the duplicates were removed. **I and II (consensus):** From both the methods, the consensus of the predicted HPIs was taken and duplicates were removed. **Interolog (unique):** The unique HPIs containing the interactions only from the interolog-based method. **Domain (unique):** The unique HPIs containing the interactions only from the domain-based method.

**Table 2:** Predicted PPIs in *T. aestivum*–*T. laevis* interactome.

Interaction Database	Number of Interactions	Number of Host Proteins	Number of Pathogen Proteins
**Interolog-based**			
BioGRID	11,003,345	51,985	3417
DIP	1,263,028	26,726	2307
HPIDB	46,117	6669	227
IntAct	4,601,434	48,463	3183
MINT	1,278,978	22,970	2536
PHI-base	35	7	5
STRING	29,978,511	82,878	2558
**Total (Interolog) (I)**	**36,330,023**	**83,637**	**3697**
**Domain-based**			
3DID	1,151,885	27,110	2483
DOMINE	5,491,200	27,901	2502
IDDI	11,548,603	33,704	3063
**Total (Domain) (II)**	**13,642,742**	**35,591**	**3212**
I and II (combined)	44,725,200	83,941	4380
I and II (consensus)	5,247,565	30,659	2305
Interolog (unique)	31,082,458	83,634	3692
Domain (unique)	8,395,177	34,176	3164

**Total (Interolog) (I):** The predicted HPIs from all seven interolog databases were merged and duplicates were removed. **Total (Domain) (II):** The predicted HPIs from all three domain databases were merged and duplicates were removed. **I and II (combined):** The predicted HPIs from both the methods were merged and the duplicates were removed. **I and II (consensus):** From both the methods, the consensus of the predicted HPIs was taken and duplicates were removed. **Interolog (unique):** The unique HPIs containing the interactions only from the interolog-based method. **Domain (unique):** The unique HPIs containing the interactions only from the domain-based method.

## Data Availability

Not applicable.

## Supplementary Materials

The supplementary material can be downloaded at: http://biocluster.usu.edu/publications/rkataria/wheat_cb_PPIs/Suppementary_Material.
