# Supplementary material for: Deciphering the Host–Pathogen Interactome of the Wheat–Common Bunt System: A Step towards Enhanced Resilience in Next Generation Wheat
Source: Int J Mol Sci. 2022 Feb 26;23(5):2589. doi: 10.3390/ijms23052589 (PMC8910311; doi:10.3390/ijms23052589)
Supplement: Supplementary file 1 [file ijms-23-02589-s001.zip › ijms-1601032-supplementary.pdf]

Supplementary Material

Deciphering the Host-Pathogen Interactome of Wheat-Common bunt system: A Step towards Enhanced Resilience in Next Generation Wheat

There are 7 Excel files that contains the supplementary material. Due to file size limitations on the "*International Journal of Molecular Sciences*" manuscript submission website, all those 7 excel files are available for access at http://biocluster.usu.edu/publications/rkataria/wheat_PPIs/Suppementary_Material.

Thank you.
